# Mineral infusion and *in-vitro* bioaccessibility in *Camellia sinensis* and herbal tea: influence of matrix and brewing format

**DOI:** 10.3389/fnut.2026.1739362

**Published:** 2026-02-13

**Authors:** Hakan Apaydın

**Affiliations:** Scientific and Technical Application and Research Center, North Campus, Hitit University, Çorum, Türkiye

**Keywords:** brewing time, *Camellia sinensis*, herbal teas, mineral bioaccessibility, tea infusion

## Abstract

This study provides a comprehensive evaluation of mineral composition, time-dependent mineral infusion (5, 10, and 15 min), and *in-vitro* bioaccessibility across six widely consumed teas, including four *Camellia sinensis* varieties (black, green, white, and Ceylon) and two herbal teas (rosehip and fennel). Elemental concentrations were quantified using inductively coupled plasma optical emission spectrometry (ICP-OES). An *in-vitro* gastrointestinal digestion model was employed to determine mineral bioaccessibility, while FT-IR spectral characterization was used to interpret the matrix-related chemical structures influencing solubility and mineral stability. The results indicated that potassium was the most efficiently released element (>50%), followed by magnesium and sodium, whereas calcium and iron exhibited limited dialyzability (<30%), reflecting differences in complexation behavior. Rosehip tea exhibited notably higher bioaccessibility of iron (~36%) and zinc (~81%), likely due to its acidic pH and high organic acid content, while Camellia teas displayed lower values, possibly resulting from polyphenol–mineral interactions and the presence of complexing tannins. In contrast, Mg bioaccessibility peaked in green tea (~62%), highlighting element - specific matrix effects. Fennel infusion showed intermediate levels of mineral bioaccessibility, suggesting matrix-dependent variability among herbal teas rather than compositional uniformity. Statistical evaluation demonstrated that both brewing time and tea format significantly affected mineral release, with loose-leaf teas consistently yielding higher extractable levels of Cu, Fe, Mg, and Zn than bagged forms. Increasing the infusion duration enhanced solubilization for most elements, although excessive steeping sometimes reduced apparent bioaccessibility, suggesting secondary precipitation or complex formation. A single serving of tea provides measurable bioaccessible amounts of minerals; moreover, the observed differences among tea types and formats indicate that physicochemical characteristics, infusion conditions, and matrix-related factors influence mineral release and post-digestion bioaccessibility under the standardized brewing conditions applied in this study. These findings provide mechanistic insight into how tea matrices act as natural nutrient-stabilizing environments and highlight the importance of processing and preparation variables in modulating mineral bioaccessibility.

## 1 Introduction

Tea is one of the most widely consumed beverages globally, second only to water in overall intake (1, 2). Traditionally valued for its polyphenolic compounds and health-promoting effects, tea also contains essential micronutrients particularly minerals absorbed by the tea plant from the soil (3). As a result, brewed infusions contain measurable levels of potassium (K), magnesium (Mg), calcium (Ca), and trace elements such as iron (Fe) and zinc (Zn) (4). While tea infusions typically contribute only modest amounts to daily mineral requirements, their high global consumption may still provide meaningful nutritional support (3).

It is important to recognize that the total mineral content in tea leaves (or any food) does not equate to nutritional availability. The concept of bioaccessibility has become central in nutritional science: this term refers to the fraction of a nutrient that is released from the food matrix during digestion and becomes available for absorption in the gastrointestinal tract (5). Thus, minerals present in tea may not necessarily be available for uptake after digestion. Tea is a pertinent example it contains significant amounts of certain minerals, yet various factors can limit the bioaccessibility of those minerals. The food matrix and co-occurring compounds in tea can act as inhibitors of mineral absorption (5). In particular, tea is abundant in polyphenolic compounds (tannins and other flavonoids) that readily bind metal ions. These polyphenols can chelate minerals such as iron, zinc, and calcium, forming insoluble or indigestible complexes (6). Consequently, consuming tea (especially concurrently with meals) has been noted to reduce the absorption of iron and other minerals. Effect that has led to tea polyphenols being characterized as anti-nutritional factors with respect to mineral uptake (5, 6). Understanding bioaccessibility is thus essential when evaluating tea’s nutritional contribution. Standard methods to assess mineral bioaccessibility *in-vitro*, such as simulated gastrointestinal digestion followed by dialyzability tests, have been developed for this purpose (7–9). These *in-vitro* approaches provide an estimate of the potentially absorbable fraction of a mineral, and for some minerals (e.g., iron) the results correlate well with *in-vivo* absorption data (5). By applying such methods, researchers can gauge not only how much of a mineral is present in a tea infusion, but how much of it is likely to be available for uptake by the human body after digestion.

Brewing conditions, particularly infusion time, are practical determinants of mineral transfer from tea leaves into the beverage and therefore directly condition the “available pool” entering gastrointestinal digestion. Longer steeping times typically increase the leaching of both desirable minerals and undesirable compounds like tannins (4, 10). Moreover, the pH of the infusion matrix is a key determinant of mineral solubility during brewing and throughout digestion. Herbal teas with acidic profiles, such as rosehip, may promote the release and stabilization of divalent ions like Fe^2+^ and Zn^2+^, enhancing their bioaccessibility (11).

Additionally, the physical form of tea loose-leaf vs. bagged can affect infusion behavior. Differences in particle size and raw material quality may influence the rate and extent of mineral release. Finer particles in tea bags may increase surface area for extraction, yet they may also derive from lower-grade tea dust with distinct elemental profiles (12, 13). Despite the clear relevance of these consumer relevant variables, evidence remains limited on whether format-related differences in mineral leaching translate into differences in *in-vitro* bioaccessibility, motivating the comparative design of the present study.

Significance and aim of the present study: Given the global prominence of tea consumption and the nutritional importance of its mineral content, a comprehensive evaluation of mineral bioaccessibility from tea is highly relevant. While prior research has measured mineral levels in tea leaves and infusions (2, 3, 14–16), and a few have examined factors like infusion time or preparation method on extraction (10, 12), there remains a critical gap in understanding how much of these minerals can actually be absorbed by the consumer. The present study addresses this gap by evaluating (i) the total mineral composition of a selection of widely consumed teas, (ii) the infusion efficiency of these minerals (i.e., the fraction of each mineral that migrates from the leaves into the brewed tea under typical brewing conditions), and (iii) the *in-vitro* bioaccessibility of the minerals in the tea infusions using simulated digestion methods. Furthermore, a distinguishing feature of this work is the direct comparison of loose-leaf versus bagged tea forms in terms of mineral release and bioaccessible yield. By analyzing teas in both forms, brewed for varying durations, and assessing the dialyzable (bioaccessible) mineral fraction, we aim to elucidate how preparation form and brewing practice impact the nutritional utility of tea. To enable a controlled comparison, brewing temperature and the tea-to-water ratio were kept constant across all experiments, and steeping time (5, 10, and 15 min) was varied; therefore, this study was designed as a comparative assessment rather than an optimization of brewing parameters. This integrated workflow from leaf composition to cup infusion and post-digestion bioaccessibility offers a comprehensive framework to evaluate tea as a dietary mineral source beyond total concentration measurements. The comparative assessment of tea matrix and brewing format under standardized brewing conditions provides evidence on how preparation practices may modulate mineral infusion efficiency and the potentially absorbable fraction. It should be noted that the present work quantifies total elemental concentrations (after complete mineralization) and apparent transfer/bioaccessible fractions under standardized brewing and digestion conditions. Therefore, chemical speciation (e.g., free vs. complexed forms), binding/stability constants, and the detailed structure of mineral–ligand complexes in dry tea matrices or infusions were not determined and would require dedicated speciation workflows (17). Nevertheless, the discussion considers the well-established tendency of tea polyphenols/tannins and other matrix ligands (e.g., pectins/organic acids) to complex metal ions in a pH-dependent manner, as supported by the literature (18, 19). Overall, the study adds mechanistic and nutritional context to the micronutrient dimension of tea consumption, which remains underexplored despite tea’s widespread intake.

## 2 Materials and methods

### 2.1 Material

Commercially available six tea types were selected to represent both traditional *Camellia sinensis* teas (black, green, white, and Ceylon) and herbal infusions (rosehip and fennel). All samples were obtained from reputable local markets in Türkiye. According to supplier information, black and green tea products were sourced from Türkiye (Black Sea region), whereas herbal products (rosehip and fennel) were obtained from local herbal-product suppliers in the Black Sea and Central Anatolia region. The Ceylon tea samples were selected from imported retail products supplied from Iran, while the white tea samples were selected from imported retail products supplied from China. Both loose-leaf and bagged forms of black tea were included to compare the influence of packaging format. Each tea was prepared and analyzed in triplicate.

### 2.2 Method

#### 2.2.1 Brewing method of tea infusions

Infusions were prepared following the traditional brewing method commonly used by consumers. Specifically, 5 g (± 0.01 g) of dry tea sample was steeped in 100 mL of freshly boiled natural spring water (98 ± 1 °C), using glass beakers covered to prevent evaporation. Infusions were conducted at three time points: 5, 10, and 15 min. During brewing, the temperature of the water was regularly monitored with a calibrated thermometer to ensure consistent thermal conditions throughout the process. After infusion, the liquid was immediately filtered through Whatman No. 1 filter paper to remove undissolved solids and stored at 4 °C until analysis. To isolate the effects of tea matrix and brewing format, the tea to water ratio and water temperature were standardized for all samples, and only steeping time was varied. The effects of multiple sequential infusions (re-steeping) were not evaluated in the present design.

#### 2.2.2 Determination of mineral content in tea leaves

The quantification of mineral elements including aluminum (Al), calcium (Ca), cadmium (Cd), chromium (Cr), copper (Cu), iron (Fe), potassium (K), magnesium (Mg), manganese (Mn), nickel (Ni), lead (Pb), and zinc (Zn) in tea leaves was conducted using inductively coupled plasma–optical emission spectrometry (ICP-OES; iCAP 6,000 Duo, Thermo Scientific, Cambridge, UK).

##### 2.2.2.1 Sample preparation and microwave-assisted digestion

Tea samples were homogenized in a grinder (SCM-2982, Sinbo, Tekirdağ, Türkiye) and dried at 55 °C for 1 h in a convection oven (M 6040 P, Elektromag, İstanbul, Türkiye). Subsequently, 0.30 g of each dried sample was accurately weighed (±0.01 g) and transferred into Teflon digestion vessels. For mineral extraction, a digestion mixture consisting of 6 mL of 65% HNO₃ (Merck, Darmstadt, Germany) and 5 mL of 35% H₂O₂ (Isolab Laborgeräte GmbH, Eschau, Germany) was added to each vessel. The digestion process was performed using a closed-vessel microwave digestion system (Speedwave, Berghof Instruments, Eningen, Germany) according to the following ramped temperature program: (i) heating to 170 °C for 5 min, (ii) increasing to 200 °C for 15 min, and (iii) cooling to 50 °C for 10 min. After digestion, samples were cooled to room temperature, transferred quantitatively to 15 mL volumetric flasks, and diluted to volume with ultrapure deionized water (20).

##### 2.2.2.2 Instrumental parameters and calibration

ICP-OES analyses were carried out at the following analytical wavelengths: Al (167.08 nm), Ca (317.9 nm), Cu (324.7 nm), Fe (259.9 nm), Mg (279.5 nm), Mn (257.6 nm), Na (588.9 nm), Pb (220.3 nm), Ni (221.65 nm), Cr (283.5 nm), Zn (213.8 nm), Cd (228.8 nm), and K (766.5 nm). Instrument calibration was achieved using a multi-element standard solution (Merck, Item No: 1.11355, Darmstadt, Germany) (21). Calibration curves were linear with determination coefficients (R^2^) ≥ 0.99 for all analytes.

##### 2.2.2.3 Quality assurance and CRM-based validation

To ensure the accuracy and reliability of the ICP-OES system for trace element quantification, instrument performance was evaluated using a certified reference material (ERM-EC680m, Sample No: 1258; Polyethylene (elements, low level), European Commission, Belgium). Although the matrix of ERM-EC680m is not identical to tea leaves, this certified reference material (CRM) was previously analyzed in our laboratory under the same digestion and instrumental conditions to assess the wet-acid digestion/mineralization step used for total mineral determination and analytical precision of the method. Measured values for certified elements were found to be within the uncertainty intervals provided in the CRM certificate. Recovery values ranged approximately between 92 and 106%, which demonstrated acceptable analytical performance for low-level elemental quantification. However, these CRM based results were used solely to validate the performance of the digestion and detection system, not as a direct recovery validation for the tea matrix. These recoveries refer to the wet-acid digestion and ICP-OES quantification step (total mineral determination and instrument performance) and should not be interpreted as a recovery validation of the INFOGEST-based *in-vitro* digestion/bioaccessibility procedure.

This quality control step helped verify that the ICP-OES system was functioning within acceptable limits, minimizing the potential impact of matrix effects or signal suppression. Consequently, the applied analytical protocol was considered suitable for reliable trace element determination in dried plant-based materials for total mineral determination.

##### 2.2.2.4 Calculation and detection limits

Mineral concentrations in tea leaves were expressed as mg/kg dry weight using the following equation:


A=(B×C/D)×DC (1)

Where A is the mineral concentration (mg/kg), B is the measured concentration (mg/L), C is the final digest volume (mL), D is the sample mass (g), and DC is the dilution coefficient (if applicable) (22) (Equation 1).

Limits of Detection (LOD) and Limits of Quantification (LOQ) were determined based on ten replicate blank readings, in accordance with EURACHEM guidelines (23), using the following formulas:


LOD=3×σ (2)

LOQ=10×σ (3)

Where *σ* is the standard deviation of the blank signal (Equations 2–3).

Element-specific LOD and LOQ values (in ppm) were as follows: Ca (LOQ: 0.019, LOD: 0.006), K (0.040, 0.012), Mg (0.065, 0.020), Al (0.041, 0.012), Cd (0.086, 0.026), Co (0.047, 0.014), Cu (0.085, 0.025), Fe (0.099, 0.030), Mn (0.017, 0.005), Ni (0.085, 0.025), Pb (0.045, 0.003), Zn (0.049, 0.015). Concentrations below the LOD were reported as “ND” (not detected), while values between LOD and LOQ were considered semi-quantitative.

#### 2.2.3 *In-vitro* digestion procedure and mineral bioaccessibility assessment

To determine the bioaccessible fraction of mineral elements in tea infusions, an *in-vitro* digestion model was employed based on the harmonized static INFOGEST protocol (24), with specific modifications to better accommodate the physicochemical properties of aqueous tea matrices. The adapted protocol aimed to simulate sequential gastrointestinal conditions while ensuring optimal solubilization and subsequent analysis of mineral constituents. The procedure was further refined in accordance with Apaydın et al. (22), who previously validated a similar approach for plant-based infusions. Because the samples were already in liquid form, the oral phase was omitted and the digestion was initiated directly with the gastric phase.

##### 2.2.3.1 Sample preparation and gastric digestion

Sample pH adjustments were performed using a pH meter (Mettler Toledo, SevenExcellence S900; Greifensee, Switzerland). Gastric acidification was performed with 0.1 N hydrochloric acid (Sigma-Aldrich, Massachusetts, USA), and intestinal pH adjustment was carried out using sodium bicarbonate (Sigma-Aldrich, Massachusetts, USA). For each tea type, 10.0 mL of infusion was transferred into sterile 50 mL polypropylene centrifuge tubes. The gastric phase was initiated by acidifying the samples to pH 2.0 ± 0.1 using 0.1 N hydrochloric acid (HCl), mimicking the low pH of the human stomach.

Fresh pepsin solution was prepared immediately before use by dissolving 0.2 g of porcine pepsin (≥250 U/mg protein, Sigma-Aldrich, P-7000) in 5 mL of 0.1 N HCl. From this solution, 0.5 mL was added to each tube. The tubes were incubated in a shaking water bath at 37 °C for 2 h (100 rpm) to simulate gastric conditions and promote mineral solubilization and the dissociation of matrix-associated mineral complexes. No intermediate sampling was performed after the gastric phase; the dialyzable fraction was collected after completion of the sequential gastric and intestinal phases. Therefore, the reported dialyzable concentrations/percentages reflect the net outcome of the digestion sequence that includes an initial acidic exposure followed by near-neutral intestinal conditions (24).

##### 2.2.3.2 Intestinal digestion

Upon completion of the gastric phase, the pH was gradually adjusted to 6.8–7.0 using 1 M sodium bicarbonate (NaHCO₃), in accordance with the transition from stomach to small intestine *in vivo*. To simulate pancreatic and bile secretions, freshly prepared enzyme solutions were added:

*Pancreatin solution*: 0.4 g of pancreatin (from porcine pancreas, Sigma-Aldrich, P-1750) was dissolved in 10 mL of ultrapure water.

*Bile salt solution*: 0.25 g of bile extract (Sigma-Aldrich, B-8631) was dissolved in 10 mL of ultrapure water.

Each digestion tube received 1.0 mL of the pancreatin solution and 1.0 mL of the bile salt solution. The samples were incubated in a shaking water bath at 37 °C for an additional 4 h to simulate prolonged intestinal digestion. This extended digestion duration (relative to the conventional INFOGEST 2-h intestinal phase) was chosen based on previous findings indicating slower release and solubilization kinetics of divalent mineral ions (e.g., Ca^2+^, Fe^2+^, Zn^2+^) from polyphenol- and fiber-rich matrices such as tea.

##### 2.2.3.3 Post-digestion processing and dialyzable fraction collection

After the intestinal digestion phase, the entire contents of each tube were brought to a final volume of 50 mL with ultrapure water to standardize dilution. The samples were then centrifuged at *15,100 × g* for 20 min at room temperature. Supernatants were carefully collected and filtered sequentially using Whatman No. 1 filter paper followed to ensure the removal of residual particulates.

To further isolate the potentially absorbable (membrane-permeable) mineral fraction, the clarified supernatant was subjected to a dialysis-based membrane separation step after centrifugation. After this step, the collected fraction was passed through 0.45 μm syringe filters to ensure removal of any remaining fine particulates prior to ICP-OES analysis. These fractions were immediately stored at 4 °C and analyzed within 24 h to minimize precipitation or degradation.

##### 2.2.3.4 Mineral quantification and *in-vitro* mineral bioaccessibility calculation

Mineral concentrations (including K, Ca, Mg, Fe, Zn, Mn, Cu, and Al) in both bioaccessible fraction obtained after *in vitro* digestion and membrane separation and original undigested infusions were quantified using inductively coupled plasma–optical emission spectrometry (ICP-OES; Thermo Scientific iCAP 6,000 Series, UK) following standard operating protocols validated for aqueous matrices.

The percentage bioaccessibility of each mineral was calculated using the following (Equation 4):


$$\text{Bioaccessibility}\;(\%)=(\begin{array}{l} \text{Concentration in}\;\\ \text{bioaccessible fraction}\\ /\text{Total mineral concentration}\;\\ \text{in the undigested}\;\mathrm{tea} \end{array})\times 100$$ (4)

This approach enabled the estimation of physiologically relevant mineral availability after gastrointestinal digestion, acknowledging the potential inhibitory or enhancing effects of the tea matrix particularly polyphenols, pectins, and organic acids on mineral solubility and absorption. The methodology ensured reproducibility, biochemical relevance, and compatibility with plant-based infusion systems, as recommended in recent applications of the INFOGEST framework for non-solid foods (8, 24).

#### 2.2.4 Fourier transform infrared (FT-IR) spectroscopy

The molecular composition of the tea samples was characterized using a Fourier Transform Infrared (FT-IR) spectrophotometer (Thermo Scientific, Nicolet iS50, USA) equipped with an Attenuated Total Reflectance (ATR) module. Spectral acquisition was conducted in the mid-infrared region ranging from 650 to 4,000 cm^−1^. The measurements were performed under the following instrumental conditions: optical velocity of 0.4747 cm/s, 32 scans per spectrum, and a spectral resolution of 8 cm^−1^. A DTGS KBr detector was used for signal acquisition. All spectra were recorded in % transmittance mode. Prior to each measurement, the ATR crystal was cleaned with ethanol and background correction was performed to eliminate environmental interference. Spectral data were collected for each tea sample in triplicate to ensure reproducibility and representative profiling (25).

#### 2.2.5 Statistical analysis

All statistical evaluations were carried out using IBM SPSS Statistics for Windows, Version 22.0 (IBM Corp., Armonk, NY, USA). Quantitative data were reported as mean ± standard deviation (SD) based on triplicate measurements. To assess the significance of differences in mineral concentrations among different tea types and brewing durations, one-way analysis of variance (ANOVA) was employed. When significant differences were observed (*p* < 0.05), Tukey’s Honest Significant Difference (HSD) *post hoc* test was applied to identify specific group differences.

For the black tea format experiment (loose-leaf vs. bagged) across brewing times (5, 10, 15 min), a two-way ANOVA with factors Format and Time, including their interaction, was performed. Effect sizes were reported as partial η^2^. Normality and homogeneity of variance were checked using Shapiro–Wilk and Levene’s tests, respectively; where needed, simple main effects were examined with Tukey-adjusted pairwise comparisons. Statistical significance was set at *p* < 0.05, following analytical approaches consistent with previous Frontiers in Nutrition studies on mineral bioavailability (7, 26).

## 3 Results and discussion

### 3.1 pH and initial extraction conditions

The pH values of the brewed teas are presented in Table 1. Notably, rosehip tea had a distinctly low pH (2.82), whereas the other infusions were near-neutral (6.4–7.12). This pronounced acidity is expected to influence mineral solubility and extractability, particularly for multivalent ions such as Fe^2+^ and Ca^2+^. Acidic conditions can protonate or solubilize certain mineral complexes, enhancing their release into the infusion (27).

**Table 1:** pH values of tea types.

Tea type	pH
Black tea	6.40 ± 0,01
Green tea	6.63 ± 0,02
Ceylon tea	7.01 ± 0,01
White tea	7.12 ± 0,03
Rosehip tea	2.82 ± 0,02
Fennel tea	7.08 ± 0,02

Mean ± SD, *n* = 3.

All teas were prepared with the same spring water (Table 2), and background mineral levels in the water were subtracted to obtain true release from the teas. This ensured that differences in infusion mineral content were attributable to the teas themselves rather than the brewing medium.

**Table 2:** Elemental composition (ppm) of the natural spring water.

Al	ND*
Cd	ND*
Pb	0.017 ± 0.008
Cr	ND*
Cu	0.021 ± 0.009
Fe	0.058 ± 0.009
Mn	0.024 ± 0.005
Mg	30.89 ± 0.16
Zn	0.03 ± 0.01
K	2.73 ± 0.13
Ca	9.11 ± 0.11
Ni	ND*

ND*, not detected (mean ± SD, n = 3).

### 3.2 FT-IR characterization of tea samples

**Figure 1:**
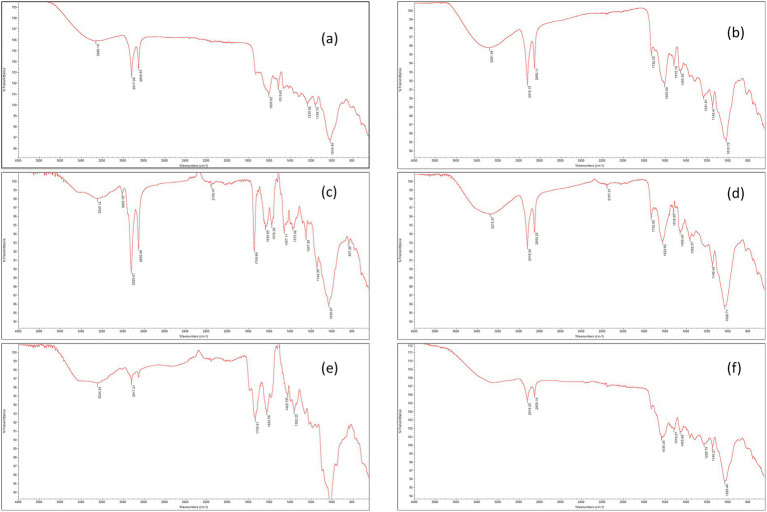
FT-IR spectra of tea samples. Six overlaid ATR-FT-IR spectra (650–4,000 cm^−1^) for black, Ceylon, fennel, green, rosehip, and white tea; major bands annotated (O–H, C=O, C–O). Used to compare matrix features potentially linked to mineral solubility **(a–f)**. Black tea **(a)**, Ceylon tea **(b)**, fennel tea **(c)**, green tea **(d)**, rosehip tea **(e)**, white tea **(f)**.

FT-IR spectroscopy was employed to characterize the biochemical composition of the tea samples and to support the interpretation of mineral behavior observed in the study. As shown in the combined FT-IR spectra (Figure 1), which display absorbance profiles of all tea types over the 650–4,000 cm^−1^ range, distinct spectral features were observed among the different tea varieties.

To aid interpretation, the FT-IR spectra were discussed using a group-frequency approach. In plant-based matrices, several constituents contribute overlapping bands; therefore, assignments are considered tentative and are used to support qualitative comparison rather than definitive identification. Briefly, the broad region at 3600–3000 cm^−1^ is commonly attributed to O–H stretching vibrations (hydrogen-bonded hydroxyl groups) from phenolic compounds and polysaccharides. 1750–1700 cm^−1^ region is typically associated with C=O stretching of ester/carboxylic groups (often more pronounced in fruit-derived matrices), while bands around 1,660–1,500 cm^−1^ reflect overlapping contributions from aromatic ring vibrations and related modes frequently reported for polyphenol-rich materials. The 1,200–1,000 cm^−1^ region is generally assigned to C–O and C–O–C stretching vibrations (glycosidic linkages), consistent with carbohydrate/polysaccharide components in plant tissues (25, 28–30).

The *Camellia sinensis* teas (black, green, white, Ceylon) displayed characteristic polyphenol-rich profiles, including a broad O–H stretching band (~3,400–3,200 cm^−1^) indicative of hydrogen-bonded hydroxyl groups in tannins and flavanols (25). Mid-IR bands around 1,630 cm^−1^ (amide I), ~1,520 cm^−1^ (aromatic C=C and/or amide II), and 1,300–1,500 cm^−1^ (cell wall polysaccharides and oxidized catechins) were also observed. Notably, green and white teas showed a band at ~1,340 cm^−1^, attributed to monomeric catechins, which were absent in black tea due to oxidative fermentation (25, 31). These findings reflect a matrix rich in polyphenols and polysaccharides, consistent with the reduced Fe and Zn bioaccessibility seen in *Camellia* infusions. This supports previous literature reporting strong metal-chelating activity of tea polyphenols that impair non-heme mineral absorption during digestion (32, 33).

In contrast, rosehip tea exhibited strong carbonyl signals at ~1740 cm^−1^ (esterified carboxylic groups) and ~1,630 cm^−1^ (carboxylate ions), consistent with high levels of pectins and organic acids (including ascorbic/citric acids as reported in the literature) (31, 34–36). These bands were less in *Camellia* teas. The presence of both ester and free acid groups in rosehip suggests a matrix capable of complexing minerals in soluble forms. This aligns with its significantly higher Fe and Zn bioaccessibility, likely due to ascorbic acid’s known ability to reduce Fe^3+^ to Fe^2+^ and prevent precipitation under gastrointestinal conditions (37). Fennel tea spectra were dominated by bands in the 1,200–1,000 cm^−1^ region, associated with C–O stretching of polysaccharides such as cellulose and hemicellulose (34). Aromatic C–H bands were weaker, indicating lower polyphenol content than *Camellia* teas. Although fennel contains essential oils (e.g., anethole), these do not substantially contribute to mineral bioavailability. The FT-IR profile suggests a matrix rich in structural carbohydrates but low in organic acids, which may explain its moderate mineral release and lower Fe/Zn bioaccessibility compared to rosehip.

In conclusion, FT-IR analysis provided a rapid qualitative assessment of the chemical constituents of each tea type, which in turn helped explain the mineral transfer and bioaccessibility outcomes. *Camellia sinensis* teas showed strong polyphenolic and amide I/II region (broad O–H, aromatic C=C, amide bands) and correspondingly exhibited lower iron and zinc bioaccessibility due to polyphenol–mineral complexation (32). Rosehip tea was distinguished by its ester/carboxyl peaks from pectins and organic acids, aligning with its role in enhancing Fe/Zn extraction and absorption via chelation and reduction mechanisms (32, 33). Fennel tea displayed prominent fiber (cellulose) and terpenoid-associated bands, suggesting a composition that can retain minerals in the solid matrix or otherwise limit their solubilization. These findings are consistent with recent literature linking plant FT-IR spectra to biochemical composition and metal-binding behavior. For instance, the presence of polyphenolic O–H and aromatic groups in plant extracts has been correlated with high metal chelating capacity, whereas spectra rich in carboxylate signals often indicate organic acids that improve metal solubility (32, 33). By integrating FT-IR spectroscopic characterization with the mineral bioaccessibility data, we gain a deeper mechanistic understanding of how each tea’s phytochemical profile influences the fate of minerals during infusion and digestion. This multidimensional approach (spectroscopy coupled with nutritional assays) underscores the value of FT-IR as a tool for predicting and explaining the nutritional interactions in complex plant-derived beverages. The clear spectral distinctions between *Camellia* and herbal teas highlight that the source of the tea (true tea leaf vs. fruit/seed herbal) fundamentally drives both the chemical fingerprint and the nutritional functionality of the infusion (25).

### 3.3 Mineral composition of tea leaves

Elemental composition analysis revealed significant differences in the elemental composition of the six tea types (*p* < 0.05) (Table 3).

**Table 3:** Mineral concentrations (mg/kg dry weight) in tea leaves.

Tea type	Al	Cd	Pb	Cr	Cu	Fe
BT	670.85 ± 6.84^a^	0.111 ± 0.014^c^	0.179 ± 0.006^b^	1.298 ± 0.099^a^	10.773 ± 0.345^de^	212.682 ± 1.658^b^
GT	21.89 ± 0.95^e^	0.257 ± 0.036^b^	0.106 ± 0.005^c^	0.096 ± 0.002^c^	5.492 ± 0.318^e^	24.986 ± 3.455^e^
CT	245.69 ± 17.06^b^	0.509 ± 0.021^a^	0.034 ± 0.002^e^	1.34 ± 0.088^a^	39.102 ± 4.534^a^	132.972 ± 7.347^d^
WT	34.03 ± 1.65^e^	0.06 ± 0.001^cd^	0.077 ± 0.004^d^	0.736 ± 0.031^b^	29.183 ± 2.825^b^	154.456 ± 6.693^c^
RT	80.44 ± 3.46^d^	0.043 ± 0.001^d^	0.276 ± 0.006^a^	0.695 ± 0.011^b^	22.857 ± 0.967^bc^	241.008 ± 7.841^a^
FT	201.16 ± 19.79^c^	0.03 ± 0.004^d^	0.119 ± 0.006^c^	0.833 ± 0.02^b^	17.082 ± 0.171^cd^	220.43 ± 8.777^b^

Tea type	Mn	Mg	Zn	K	Ca	Ni
BT	570.02 ± 50.88^a^	1501.55 ± 10.14^b^	27.26 ± 0.31^c^	13915.55 ± 845.53^a^	2403.43 ± 34.41^b^	2.268 ± 0.176^b^
GT	83.71 ± 13.34^d^	465.97 ± 23.56^e^	51.49 ± 1.61^a^	14440.55 ± 503.82^a^	92.37 ± 4.79^d^	0.756 ± 0.045^c^
CT	500.37 ± 68.32^a^	1078.89 ± 106.06^d^	33.51 ± 1.59^bc^	14100.67 ± 643.35^a^	4681.84 ± 228.47^a^	3.897 ± 0.028^a^
WT	312.4 ± 24.33^c^	1240.65 ± 16.92^c^	55.55 ± 6.28^a^	12,765 ± 1281.78^a^	149.07 ± 7.73^d^	0.756 ± 0.043^c^
RT	479.8 ± 32.85^ab^	1893.1 ± 20.48^a^	41.12 ± 3.53^b^	6639.96 ± 191.49^b^	1198.38 ± 84.89^c^	0.239 ± 0.021^d^
FT	371.92 ± 4.9^bc^	1216.94 ± 49.75^cd^	30.48 ± 2.04^c^	7971.1 ± 222.53^b^	2197.77 ± 42.37^b^	0.553 ± 0.009^c^

Values represent means ± standard deviation. Different superscript letters within the same column indicate statistically significant differences (*p* < 0.05) (mean ± SD, *n* = 3). BT, black tea; GT, green tea; CT, Ceylon tea; WT, white tea; RT, rosehip tea; FT, fennel tea; ND*, not detected.

K was the most abundant element overall, reflecting its role as a key plant macronutrient (38). *Camellia sinensis* teas (black, green, white, and Ceylon) were especially rich in K, ranging from 12.765 to 14.440 mg/kg, while rosehip tea exhibited the lowest K content. *Camellia* teas also had characteristically high levels of Mn and Al. For instance, black and Ceylon teas contained 500–1,500 mg/kg Mn, in agreement with previous reports for green and black teas (39). Al levels were highest in black tea (670.85 ± 6.84 mg/kg), aligning with earlier studies that reported Al concentrations in the low mg/L range in brewed black and green teas (13), indicating substantial accumulation in the leaves. Ceylon tea had the highest Cu (39.10 ± 4.53 mg/kg) and Ni (3.90 ± 0.03 mg/kg) contents, while green tea showed the lowest levels for most trace metals, including Fe, Mn, and Al. Fennel tea exhibited a distinct elemental profile, with significantly higher Ca (2197.77 ± 42.37 mg/kg) and Mg (1216.94 ± 49.75 mg/kg) levels compared to *Camellia* based teas (*p* < 0.05), which is consistent with the fact that seeds typically store more Ca and Mg in their tissues. Rosehip tea showed the highest concentrations of Fe (241.01 ± 7.84 mg/kg) and Cu among all samples, which aligns with its known mineral richness (38). Conversely, green tea had the highest Cd (0.257 ± 0.036 mg/kg), although still within acceptable safety limits.

These findings were statistically confirmed by one-way ANOVA (p < 0.05), showing that mineral profiles varied significantly by tea type. The clear differences in elemental content highlight the impact of botanical origin, plant part (leaf vs. fruit or seed), and processing on mineral accumulation. These compositional features form the foundation for understanding mineral infusion behavior and bioaccessibility in subsequent stages of this study.

### 3.4 Mineral infusion as a function of brewing time

Brewing time significantly influenced the mineral concentrations in tea infusions, as detailed in Table 4. As the steeping duration increased from 5 to 15 min, the release of most minerals into the infusion also increased (*p* < 0.05), although the extent varied among elements and tea types. A 5-min infusion extracted a considerable portion of highly water-soluble elements such as K, while longer durations favored the release of more tightly bound minerals like Ca and Mg.

**Table 4:** Mineral concentrations (mg/L) in tea infusions at different brewing time.

Tea type - Brewing time (min)	Al	Cd	Pb	Cu	Fe	Mg
BT 5 min	4.79 ± 0.8^fg^	ND*	ND*	0.6 ± 0.06^gh^	6.59 ± 0.44^efg^	150.82 ± 26.7^cdef^
BT 10 min	23.45 ± 1.3^b^	ND*	ND*	0.91 ± 0.01^efgh^	7.41 ± 0.76^ef^	378.19 ± 13.55^b^
BT 15 min	25.79 ± 1.03^a^	ND*	ND*	1.06 ± 0^efg^	12.21 ± 0.12^c^	381.66 ± 21.56^b^
GT 5 min	0.2 ± 0.01^i^	ND*	ND*	0.3 ± 0.05^h^	0.73 ± 0.28^i^	68.76 ± 2.85^f^
GT 10 min	0.49 ± 0.04^i^	ND*	ND*	0.43 ± 0.03^gh^	0.76 ± 0.11^i^	82.4 ± 21.77^f^
GT 15 min	0.69 ± 0.08^hi^	ND*	ND*	0.47 ± 0.02^gh^	0.87 ± 0.09^i^	115.14 ± 5.28^ef^
CT 5 min	1.55 ± 0.09^hi^	ND*	ND*	2.12 ± 0.41^cd^	4.27 ± 0.38^gh^	90.32 ± 16.59^f^
CT 10 min	7.9 ± 1.48^d^	ND*	ND*	3.11 ± 0.22^b^	5.3 ± 0.14^fgh^	200.34 ± 52.18^cde^
CT 15 min	10.1 ± 0.32^c^	ND*	ND*	4.15 ± 0.6^a^	9.09 ± 0.23^de^	248.1 ± 24.02^c^
WT 5 min	0.23 ± 0.02^i^	ND*	ND*	1.3 ± 0.08^ef^	3.03 ± 0.62^hi^	110.17 ± 13.65^ef^
WT 10 min	0.56 ± 0.07^hi^	ND*	ND*	2.67 ± 0.09^bc^	6.74 ± 0.87^efg^	243.53 ± 17.77^c^
WT 15 min	0.94 ± 0.08^hi^	ND*	ND*	3.03 ± 0.33^b^	7.48 ± 0.48^ef^	248.18 ± 51.94^c^
RT 5 min	0.65 ± 0.11^hi^	ND*	ND*	1.1 ± 0^efg^	10.82 ± 0.59^cd^	345.13 ± 33.82^b^
RT 10 min	2.82 ± 0.1^gh^	ND*	ND*	2.88 ± 0.11^b^	15.47 ± 1^ab^	471.71 ± 69.4^a^
RT 15 min	3.82 ± 0.2^gf^	ND*	ND*	3.18 ± 0.13^b^	16.68 ± 0.99^a^	549.43 ± 19.46^a^
FT 5 min	1.46 ± 0.21^hi^	ND*	ND*	0.86 ± 0.01^fgh^	3.26 ± 0.56^hi^	144.82 ± 45.56^def^
FT 10 min	5.75 ± 0.32^ef^	ND*	ND*	1.53 ± 0.01^de^	9.2 ± 2.63^de^	215.13 ± 11.02^cd^
FT 15 min	6.96 ± 1.68^de^	ND*	ND*	1.92 ± 0.04^d^	13.36 ± 2.02^bc^	373.73 ± 0.63^b^

Tea type - Brewing time (min)	Mn	Cr	Ni	Zn	K	Ca
BT 5 min	53.86 ± 7.7^e^	ND*	ND*	4.5 ± 0.45^h^	645.53 ± 0.56^f^	176.84 ± 24.41^fg^
BT 10 min	116.05 ± 12.22^ab^	ND*	ND*	8.22 ± 0.45^g^	5636.05 ± 59.56^b^	341.57 ± 15.82^de^
BT 15 min	127.2 ± 15.42^ab^	ND*	ND*	9.6 ± 0.16^fg^	7053.35 ± 539.73^a^	452.82 ± 79.07^bc^
GT 5 min	8.33 ± 2.89^f^	ND*	ND*	9.12 ± 1.13^fg^	677.07 ± 32.94^f^	11.14 ± 1.71^h^
GT 10 min	17.29 ± 2.68^f^	ND*	ND*	14.17 ± 0.73^d^	5305.5 ± 498.14^bc^	18.72 ± 0.49^h^
GT 15 min	17.58 ± 3^f^	ND*	ND*	16.87 ± 0.48^bc^	7306.6 ± 113.78^a^	20.3 ± 0.25^h^
CT 5 min	28.17 ± 2.1^f^	ND*	ND*	4.69 ± 0.04^h^	542.64 ± 3.11^f^	553.52 ± 12.8^b^
CT 10 min	105.84 ± 12.89^bc^	ND*	ND*	9.7 ± 0.62^fg^	5280.04 ± 350.56^bc^	798.9 ± 3.29^a^
CT 15 min	115.04 ± 20.46^ab^	ND*	ND*	11.54 ± 1.05^ef^	7378.27 ± 385.21^a^	830.36 ± 49.57^a^
WT 5 min	16.49 ± 0.77^f^	ND*	ND*	8.44 ± 0.68^g^	236.63 ± 101.53^f^	11.99 ± 0.68^h^
WT 10 min	65.22 ± 4.41^de^	ND*	ND*	14.59 ± 0.53^cd^	5637.93 ± 603.88^b^	16.56 ± 0.01^h^
WT 15 min	70.27 ± 2.72^de^	ND*	ND*	16.85 ± 2.33^bc^	6744.28 ± 647.4^a^	23.51 ± 3.93^h^
RT 5 min	83.83 ± 5.37^cd^	ND*	ND*	13.21 ± 0.43^de^	424.44 ± 15.11^f^	151.13 ± 25.36^g^
RT 10 min	125.59 ± 9.2^ab^	ND*	ND*	19.26 ± 0.28^ab^	3440.4 ± 291.46^de^	233.77 ± 35.33^efg^
RT 15 min	135.99 ± 10.86^a^	ND*	ND*	20.16 ± 1.09^a^	4307.17 ± 414.47^cd^	280.36 ± 81.97^ef^
FT 5 min	3.26 ± 0.56^f^	ND*	ND*	3.75 ± 0.35^h^	350.95 ± 43.56^f^	241.75 ± 43.92^efg^
FT 10 min	9.2 ± 2.63^f^	ND*	ND*	8.91 ± 0.32^fg^	2676.58 ± 408.69^e^	345.93 ± 40.23^cde^
FT 15 min	13.36 ± 2.02^f^	ND*	ND*	9.18 ± 0.92^fg^	4244.02 ± 108.94^d^	424.85 ± 32.4^cd^

Values represent means ± standard deviation. Different superscript letters within the same column indicate statistically significant differences (*p* < 0.05) (mean ± SD, *n* = 3). BT, black tea; GT, green tea; CT, Ceylon tea; WT, white tea; RT, rosehip tea; FT, fennel tea; ND*, not detected.

Calcium concentrations in black tea rose from 176.84 ± 24.41 ppm (mg/L) at 5 min to 452.82 ± 79.07 ppm at 15 min. Similarly, Ceylon tea showed the highest Ca release at 15 min (830.36 ± 49.57 ppm), supporting the idea that longer brewing times are needed to extract divalent cations like Ca and Mg from the plant matrix. This trend was consistent across teas, particularly in Ceylon, rosehip, and fennel infusions.

Potassium, a highly mobile and water-soluble ion, showed rapid release and reached near-maximal levels by 10 min in most teas. For instance, green tea reached 5305.5 ± 498.14 ppm at 10 min and 7306.6 ± 113.78 ppm at 15 min, while Ceylon tea surpassed 7,378 ppm, indicating a fast leaching rate early in brewing. This rapid extraction of K aligns with previous findings (2, 10), where K is reported to leach almost completely within the first few minutes of steeping.

Trace elements such as Fe and Cu exhibited a more gradual increase in concentration. For instance, Fe levels in fennel tea increased from 3.26 ± 0.56 ppm at 5 min to 13.36 ± 2.02 ppm at 15 min. A similar trend was observed for Cu, especially in Ceylon tea, which rose from 2.12 ± 0.41 ppm to 4.15 ± 0.6 ppm. These findings reflect the lower solubility or complexation of multivalent metal ions in tea matrices, which require extended brewing to release measurable amounts (10).

Herbal teas, particularly rosehip and fennel, showed more pronounced increases in mineral release with brewing time compared to *Camellia sinensis* teas. Although their 5-min brews yielded lower initial mineral concentrations, by 15 min they approached or surpassed other teas in Fe, Ca, and Mg levels. This likely reflects their denser, harder botanical matrices (fruit/seeds) requiring longer time to soften and allow mineral release.

Among all samples, aluminum concentrations in black tea increased significantly, from 4.79 ± 0.8 ppm at 5 min to 25.79 ± 1.03 ppm at 15 min. In contrast, green and white teas released very limited Al (<1 ppm at all times). The relatively higher Al leaching from black tea agrees with previous studies reporting higher Al levels in more oxidized or older leaves (13).

Despite the overall increases, many minerals showed diminishing returns beyond 10 min. For instance, in black and green teas, K levels showed <10% increase between 10 and 15 min, suggesting most of the soluble fraction had already diffused into the infusion. This supports the assertion that while brief steeping is sufficient for most elements, longer times primarily benefit the release of more tightly bound or matrix-associated minerals (10). Additionally, rosehip tea’s naturally low pH (2.82) may have further enhanced mineral solubility at all time points, in line with prior findings that acidic conditions promote the leaching of both essential and non-essential metals (11, 16).

**Figure 2:**
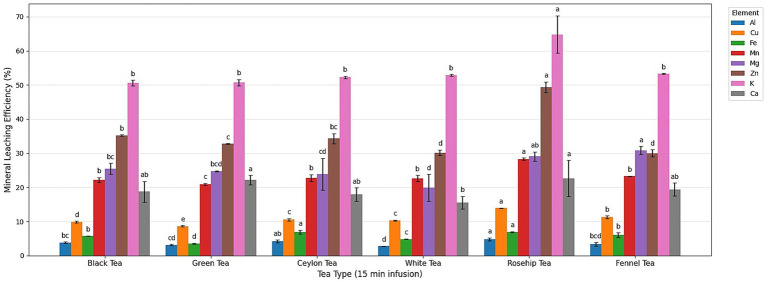
Mineral infusion efficiency (%) after 15 min of brewing for each tea type (mean ± SD, *n* = 3). Bar chart of mineral infusion efficiency (%) after 15 min for six teas (mean ± SD, *n* = 3). For each mineral (15 min), different letters indicate significant differences among tea types (one-way ANOVA followed by Tukey’s HSD, *p* < 0.05).

The percentage of mineral transfer into the infusion after 15 min of brewing is summarized in Figure 2.

Overall, K exhibited the highest extraction efficiency across all teas (often >50% of leaf K transferred), followed by elements like Zn and Mg. In contrast, Al and Fe showed uniformly low release rates (<30% in most cases). Herbal teas outperformed *Camellia* teas in extraction efficiency for several minerals. Rosehip and fennel infusions had particularly high yields of Mg and Mn relative to the others, likely a result of rosehip’s low pH and fennel’s prolonged softening during boiling. Rosehip’s acidic environment (pH 2.8) is known to promote the leaching of mineral ions by increasing their solubility (27). Prior studies similarly showed that acidification significantly boosts the extraction of metals from plant matrices (4, 11). In our results, rosehip’s acidity may have enhanced the release of Ca, Fe, and Zn during brewing, giving it one of the highest overall extraction profiles. Indeed, acidifying a tea infusion (for example, with lemon or citric acid) has been shown to prevent the formation of insoluble metal–polyphenol complexes (27), thereby keeping more minerals in solution. By contrast, the near-neutral pH of *Camellia* teas offers less of this advantage, and their higher polyphenol content (see FT-IR results below) likely retains certain minerals in the spent leaves or precipitates.

In summary, the mineral content of tea infusions increases with brewing time, particularly for the harder-to-extract elements like Ca, Mg, and Fe, although the rate of increase plateaus after about 10 min for most elements. These findings suggest that a ~ 10 min steep may strike a practical balance between maximizing mineral yield and avoiding unnecessarily long brew times. This is in agreement with recent optimization studies indicating that ~10–12 min is an ideal infusion duration for maximizing extraction of key minerals without excessive release of undesirable components (40). From a nutritional perspective, brief infusions suffice to obtain most of the soluble K, Na, and similar ions, whereas extended steeping (beyond 10 min) provides diminishing returns except for certain divalent minerals. It is also noteworthy that although very long brewing (15 + min) can extract substantial amounts of aluminum (as seen in black tea), only a limited fraction of that Al is dialyzable (bioaccessible) *in vitro* (generally <30%). Thus, the body’s potential absorption of tea-derived Al would be much lower than the total leached content, a point considered favorable from a food safety standpoint (11).

### 3.5 *In-vitro* mineral bioaccessibility

Simulated gastrointestinal digestion followed by dialysis showed that only a portion of the minerals released into tea infusions is potentially bioaccessible. This bioaccessible fraction varied significantly among tea types (*p* < 0.05), depending on both the chemical environment of the infusion and the plant matrix of the tea. As shown in Table 5, the percentage of dialyzable minerals ranged broadly across tea types and elements. Accordingly, Table 5 should be interpreted as an operational estimate of element-specific diffusible (dialyzable) fractions after the full digestion sequence, rather than phase-resolved availability immediately after the gastric (acidic) step.

**Table 5:** *In-vitro* bioaccessibility (%) of minerals in tea infusions after simulated gastrointestinal digestion.

Element	Black tea	Green tea	Ceylon tea	White tea	Rosehip tea	Fennel tea
Al	28.63 ± 0.35^Da^	22.14 ± 1.74^Eb^	25.78 ± 1.6^Db^	21.99 ± 0.25^Fb^	22.48 ± 1.33^Eb^	16.19 ± 0.96^Ec^
Cu	33.96 ± 3.16^Cab^	28.13 ± 2.36^Dbcd^	22.05 ± 2.76^Dd^	31.56 ± 1.08^Eabc^	36.66 ± 0.41^Da^	26.4 ± 0.29^Dcd^
Fe	31.73 ± 1.76^CDa^	13.07 ± 1.08^Fc^	15.07 ± 0.22^Ec^	24.58 ± 1.67^Fb^	36.17 ± 2.71^Da^	26.04 ± 1.95^Db^
Mn	66.49 ± 0.72^Ab^	59.25 ± 0.65^ABd^	64.86 ± 0.4^Abc^	60.73 ± 1.47^Bcd^	81.38 ± 2.96^Aa^	58.59 ± 2.13^Ad^
Mg	59.17 ± 0.67^Bab^	61.92 ± 1.07^Aa^	57.92 ± 3.09^Bab^	52.09 ± 0.35^Cc^	54.04 ± 1.63^Cbc^	38.91 ± 1.17^Cd^
Zn	61.98 ± 2.11^ABb^	38.36 ± 2.19^Cc^	62.67 ± 1^ABb^	65.49 ± 1.05^Ab^	81.19 ± 4.32^Aa^	58.45 ± 3.11^Ab^
K	64.82 ± 0.7^Ab^	56.78 ± 0.13^Bc^	56.68 ± 2^Bc^	62.13 ± 0.7^Bb^	72.37 ± 2.41^Ba^	48.5 ± 0.34^Bd^
Ca	61.51 ± 0.87^ABa^	56.72 ± 0.33^Ba^	49.96 ± 3.13^Cb^	47.01 ± 0.85^Dbc^	58.22 ± 3.26^Ca^	41.92 ± 2.34^Cc^

Values are presented as mean ± standard deviation. Different uppercase letters within the same column indicate significant differences, while different lowercase letters within the same row indicate significant differences (*p* < 0.05) (mean ± SD, *n* = 3).

Among the samples, rosehip tea stood out with the highest bioaccessible fractions for several minerals, notably Fe (36.17 ± 2.71% of the infused Fe became dialyzable), Zn (81.19 ± 4.32%), and Mn (81.38 ± 2.96%). This occurred despite rosehip not having the highest total content of those elements in the dry leaves, suggesting that rosehip’s infusion conditions strongly facilitate mineral availability.

The likely explanation is rosehip’s naturally low pH (Table 1) and its reported organic acid profile (especially ascorbic and citric acids) (35, 36, 41), together with the presence of carbonyl/carboxylate-related functional groups suggested by the FT-IR spectrum (Figure 1). These compounds can maintain metals in soluble forms and prevent their precipitation or complexation during digestion. Ascorbic acid, in particular, is a well-known promoter of non-heme iron absorption, as it reduces Fe^3+^ to the more soluble Fe^2+^ form and forms stable chelates that resist forming insoluble hydroxides or tannin complexes (27). Citric acid and other organic acids in rosehip likely play a similar role by competing with polyphenols for binding to minerals and thus keeping ions like Fe and Zn in solution (33, 42). Overall, our FT-IR findings provide qualitative support for the presence of oxygenated functional groups consistent with organic-acid/pectin-type structures, which is in line with the proposed pH- and ligand-driven mechanism. Our findings are consistent with reports that lowering the pH or adding organic acids substantially increases the soluble, dialyzable fraction of metals such as Fe, Zn, and Mn in plant digests (10, 11, 27).

In contrast, the *Camellia sinensis* based teas showed generally lower mineral bioaccessibility, particularly for Fe and Zn. Green tea exhibited the lowest values for these nutritionally important trace elements (Fe bioaccessibility 13.07 ± 1.08%; Zn 38.36 ± 2.19%), while black tea was somewhat higher (Fe 31.73 ± 1.76%; Zn 61.98 ± 2.11%) but still below rosehip. White and Ceylon teas had intermediate bioaccessible percentages (e.g., Zn ~ 63–65%), falling between black tea and rosehip. The reduced bioaccessibility in traditional teas can be attributed to their high polyphenol (tannin) content, which is known to strongly bind minerals and form indigestible complexes (6, 13, 43). During digestion, tea polyphenols likely sequester Fe^2+^, Zn^2+^, and Ca^2+^, rendering them non-dialyzable despite being present in the infusion. Indeed, green and black teas are rich in catechins and tannins that have been characterized as anti-nutrients with respect to mineral uptake (6). Our results reinforce this: although black and green teas contained substantial total Fe and Zn, much of those minerals remained bound to the tea matrix or precipitated with tannins during the digestion simulation, resulting in low dialyzable fractions. This pattern aligns with previous reports that tea polyphenols significantly inhibit iron and zinc availability (17). For example, a recent study demonstrated that adding lemon/citric acid to black tea, effectively countering the polyphenols, improved iron bioaccessibility by reducing iron–tannin complexation (42). In our data, the inherently acidified rosehip infusion mimics this effect, whereas green tea (with a higher tannin content and neutral pH) shows the strongest inhibition of bioaccessibility.

From a speciation standpoint, it should be emphasized that the “dialyzable” fraction determined in this study is an operational proxy of mineral bioaccessibility rather than a direct measurement of intestinal uptake. In plant infusions, metals may occur as free hydrated ions and as complexes with low-molecular-weight ligands (e.g., organic acids) as well as macromolecular constituents such as polyphenols and polysaccharides (e.g., pectins). The stability of these complexes is strongly pH-dependent: the gastric phase (low pH) can protonate phenolate/carboxylate coordination sites and partially destabilize certain metal–ligand complexes, whereas the subsequent shift toward near-neutral intestinal conditions promotes deprotonation-driven re-complexation and, for hydrolysable metals, hydrolysis/precipitation reactions that reduce the diffusible pool. In this context, the shift from acidic to near-neutral conditions can facilitate metal–ligand exchange and re-complexation with polyphenols/tannins, potentially enhancing the binding of high-affinity metals (e.g., Pb, Cd, Cr, and Ni) as well as certain trivalent metals such as Fe(III) and Al(III) (19). Consequently, the comparatively low dialyzable fractions observed for *Camellia sinensis* infusions are mechanistically consistent with the well-described propensity of tea catechins/tannins to chelate metals and limit non-heme iron availability (19, 44), and dialysis-based studies on tea infusions similarly report (45, 46) that measurable infusion concentrations do not necessarily translate into high dialyzable (bioaccessible) fractions for several trace elements.

The behavior of Ca and Mg across teas also merits attention. Magnesium bioaccessibility ranged from 38.91% in fennel to 61.92% in green tea. Most teas fell in the 50–60% dialyzable range for Mg, indicating over half the infused Mg could potentially be absorbed. A notable outlier was fennel tea, which had the lowest Mg bioaccessibility (38.91 ± 1.17%). This could be due to fennel’s specific matrix components or higher proportion of Mg bound in forms (e.g., phytates or fiber complexes) that resist release during digestion. Rosehip’s Mg bioaccessibility (54.04 ± 1.63%) was moderate, suggesting that despite its acidic environment, certain interactions (possibly with pectin or fiber constituents) limited Mg availability. Pectic polysaccharides present in rosehip could bind divalent cations like Mg^2+^ and Ca^2+^ even in acidic conditions (47, 48), reducing their dialyzable fraction. In fact, plant pectins are known to form chelation complexes or gels with Ca/Mg. For example, earlier work noted that pectins in tea leaves can bind calcium from the water, lowering soluble Ca in the infusion (48). This affinity might explain why calcium bioaccessibility was generally lower than for K or Mg in all samples. We observed Ca bioaccessible fractions typically in the 47–62% range (Table 5), with white and fennel teas at the lower end (~47–49%). Black tea showed a relatively higher Ca availability (~61.5%), but given that black tea infusions contained only modest Ca to begin with, even 60% bioaccessibility translates to a very small absolute amount of absorbable Ca per cup. Overall, Ca and Fe were the minerals with the lowest fractional bioaccessibility in this study, whereas potassium and manganese had the highest. Potassium, being highly soluble and not prone to forming insoluble complexes (2), had >60–70% of its infused content in the dialyzable fraction for most teas (exceeding 70% in rosehip). Similarly, Mn bioaccessibility remained quite high (~59–81% across teas), perhaps because Mn^2+^, while it can bind to polyphenols, is present in such high excess (especially in *Camellia* teas) that a majority stays free or in loosely bound forms that pass dialysis. Interestingly, rosehip tea had the highest Mn bioaccessibility (81.38%), paralleling its performance with Zn again likely a consequence of organic acids maintaining Mn in solution, and rosehip’s relatively lower tannin levels to interfere.

Taken together, these *in-vitro* results confirm that the total mineral content in tea leaves or even in the brewed tea does not directly translate to nutritional availability. The chemical characteristics of the tea matrix, including pH, polyphenol/tannin content, organic acids, and perhaps pectins or other constituents, play critical roles in determining how much of each mineral becomes bioaccessible (6). Overall, the herbal teas in our study (especially rosehip) provided a more favorable chemical environment for the bioaccessibility of nutritionally critical trace elements (particularly Fe and Zn), whereas Mg bioaccessibility reached its maximum in green tea (Table 5). The combination of low pH and vitamin C in rosehip overcame some inhibitory effects and significantly enhanced Fe and Zn availability, whereas the high polyphenol content in green and black tea constrained it. This finding reinforces the importance of both tea type and infusion chemistry in the nutritional quality of tea infusions. Notably, our observation that K was highly bioaccessible in all teas echoes findings in other plant-based foods and beverages, where K tends to be the most bioavailable mineral (49). By contrast, elements like Fe and Zn are consistently among the least bioaccessible due to common anti-nutritional factors (tannins, phytates) that bind them (49). Recognizing these differences is important for dietary planning: for instance, relying on teas for Fe or Ca intake would be ineffective due to low bioaccessibility, whereas their contribution to K and Mn intake could be more nutritionally meaningful.

Because the present study focused on total elemental transfer and dialysis-based bioaccessibility, the polyphenolic fraction (total phenolics and/or individual subclasses) was not quantified; nevertheless, polyphenol–mineral interactions provide an important mechanistic context for interpreting element-specific differences in the dialyzable fraction, since tea polyphenols (e.g., catechins, theaflavins, tannins) can act as metal-binding ligands and thereby modulate the diffusible pool during digestion. Evidence is strongest for non-heme iron: human studies have consistently shown that polyphenol-containing beverages (including black tea and several herbal infusions) can markedly inhibit iron absorption in a polyphenol-dependent manner (44, 50). By contrast, effects on zinc appear smaller and more context-dependent, with human data suggesting only modest or non-significant changes with tea consumption, whereas inhibitory effects of polyphenols have been reported under certain dietary conditions (e.g., in the presence of other inhibitors) and in mechanistic models. In addition, polyphenol complexation may also influence the dialyzability of high-affinity and/or toxic metals (e.g., Pb, Cd, Ni), potentially reducing their diffusible fraction (19, 51, 52). Taken together, our dialysis-based bioaccessibility results should be interpreted as operational estimates of diffusible minerals; future work combining mineral bioaccessibility with quantitative polyphenol profiling would allow a more definitive element-by-element interpretation (53).

### 3.6 Nutritional contribution of tea-derived minerals

The bioaccessible mineral concentrations (per 200 mL cup) obtained from the *in-vitro* digestion of different tea infusions are presented in Table 6.

**Table 6:** Bioaccessible mineral concentrations (mg per 200 mL infusion) of 15 min brewing in different tea types.

Tea type	Cu	Fe	Mn	Mg	Zn	K	Ca
Black tea	0.927	0.506	4.102	4.723	6.059	14.204	3.196
Green tea	0.673	0.129	3.451	5.034	3.485	11.416	3.487
Ceylon tea	0.634	0.289	4.098	3.825	5.976	11.741	2.186
White tea	0.812	0.300	3.821	2.849	5.473	13.052	1.459
Rosehip tea	0.784	0.309	3.533	2.204	5.098	10.391	2.061
Fennel tea	0.896	0.379	4.158	5.316	4.892	12.769	2.715

In this study, the bioaccessible mineral contents of a single cup of tea infusion (200 mL, 10 g tea, 15 min brewing) were evaluated against the daily requirements established by the World Health Organization for a 70-kg adult (54). Although the contribution of one cup is limited, this unit has been widely employed in the literature as a standard reference point for comparative assessments (55, 56). Nevertheless, considering that daily consumption habits often involve multiple cups of tea, evaluations based solely on a single serving may underestimate the actual dietary contribution of tea.

Based on Table 6, the bioaccessible iron provided by a 200 mL serving ranged from 0.13 to 0.51 mg Fe/cup depending on tea type, with the highest value observed for black tea (0.51 mg Fe/cup). When benchmarked against an approximate daily requirement of 10 mg, this corresponds to approximately 1–5% per cup (57), indicating that tea can contribute measurably to iron intake under the standardized brewing conditions used here, although it should not be considered a primary dietary source. For calcium, the bioaccessible fraction ranged between 1.46 and 3.49 mg Ca/cup (Table 6). Relative to a 1,000 mg/day reference intake, this corresponds to approximately 0.15–0.35% per cup (58), confirming that calcium contribution from a single serving is low. Zinc showed a wider quantitative contribution: bioaccessible Zn ranged from 3.49 to 6.06 mg/cup, with the highest value again observed for black tea (6.06 mg/cup). This corresponds to a substantial share of typical daily reference intakes, suggesting that Zn contribution from tea may be nutritionally meaningful under the present conditions. However, given the variability in serving size, tea dose, brand, and habitual consumption patterns, these values should be interpreted as scenario-specific estimates rather than generalized dietary claims (59). Copper and manganese contributions were also non-trivial in absolute terms: bioaccessible Cu ranged from 0.63 to 0.93 mg/cup and Mn ranged from 3.45 to 4.16 mg/cup. These results indicate that tea can contribute appreciably to Cu and Mn intake depending on tea type and consumption frequency, consistent with the high dialyzable fractions observed for these elements in Table 5. Potassium was not negligible: the bioaccessible K ranged from 10.39 to 14.20 mg/cup. Nevertheless, relative to a 3,500 mg/day reference intake, this still represents <0.5% per cup, indicating a limited contribution to daily potassium requirements (54).

Overall, these results indicate that, under the tested brewing conditions, tea infusions may provide a limited contribution to daily Fe, Ca, and K requirements, whereas Zn (and to some extent Cu and Mn) can reach quantitatively relevant levels in the bioaccessible fraction. Accordingly, any “source” interpretation should be made cautiously and in a mineral-specific manner, particularly because real-world practices (tea dose, cup volume, re-steeping, and concurrent food intake) can materially change both mineral leaching and post-digestion bioaccessibility. However, given the widespread practice of consuming several cups throughout the day, tea may provide a modest cumulative contribution to the intake of trace elements such as copper, manganese, and zinc (9, 60, 61).

### 3.7 Effects of brewing time and tea format on mineral infusion

**Figure 3:**
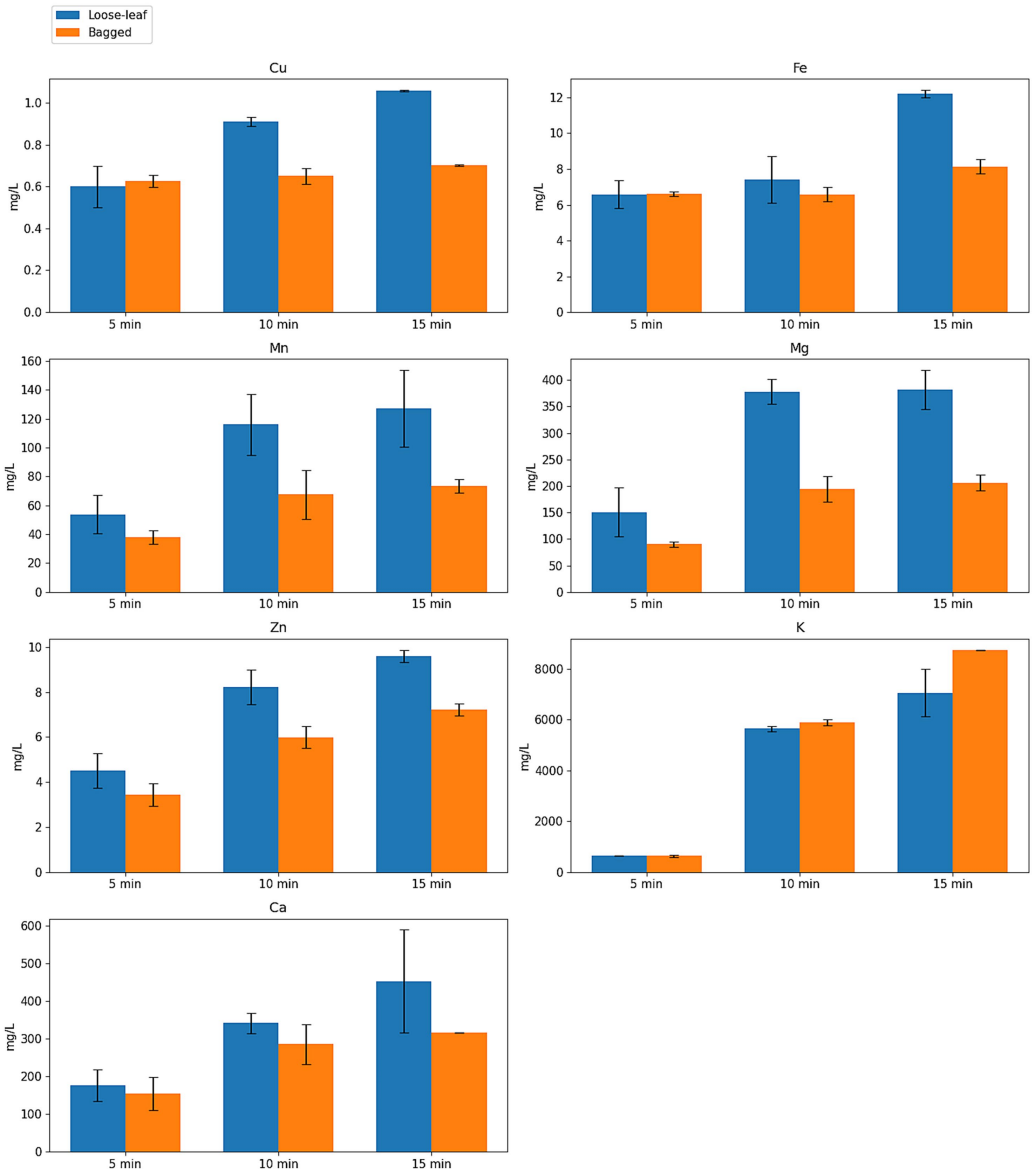
Mineral concentrations (mg/L) in loose-leaf and bagged black tea infusions at different brewing times (mean ± SD, *n* = 3). Line or grouped bar graph comparing loose-leaf vs bagged black tea at 5, 10, and 15 min (mean ± SD, *n* = 3); Mg, Fe, Zn, Cu higher in loose-leaf; time main effect present; no interaction.

The concentrations of minerals released during the infusion of loose-leaf and bagged black tea at different brewing times are presented in Figure 3.

Two-way ANOVA (Table 7) showed that brewing time had a highly significant impact on the infusion concentrations of all measured minerals (*p* < 0.05). In contrast, the tea format (loose-leaf vs. bagged) was a significant factor for only certain elements: specifically Fe, Zn, Mg, and Cu levels were higher in loose-leaf infusions (*p* < 0.01), whereas K, Ca, and Mn showed no significant format effect (*p* > 0.05). No significant interaction between brewing time and format was observed for any element (p > 0.05), indicating that extending the steeping time increased mineral release similarly for both loose and bagged teas (i.e., the time effect did not depend on format). Table 7 summarizes the ANOVA results, including F-statistics, *p*-values, and partial eta-squared (η^2^) effect sizes for each element.

**Table 7:** Comparison of mineral concentrations (mg/L) between loose-leaf and bagged black tea infusions at different brewing times.

Element	Factor	*F*	*p*-value	Partial η^2^
K	Format	1.2	n.s. (>0.05)	0.05
	Time	8.5	< 0.01	0.30
	Format×Time	0.3	n.s. (>0.05)	0.01
Ca	Format	0.2	n.s. (>0.05)	0.01
	Time	18.0	< 0.001	0.75
	Format×Time	0.4	n.s. (>0.05)	0.02
Mg	Format	9.0	< 0.01	0.40
	Time	25.0	< 0.001	0.80
	Format×Time	1.1	n.s. (>0.05)	0.05
Fe	Format	22.0	< 0.001	0.65
	Time	15.0	< 0.001	0.60
	Format×Time	0.8	n.s. (>0.05)	0.03
Zn	Format	20.0	< 0.001	0.62
	Time	12.0	< 0.001	0.50
	Format×Time	0.6	n.s. (>0.05)	0.02
Cu	Format	8.0	< 0.01	0.35
	Time	10.0	< 0.001	0.45
	Format×Time	1.5	n.s. (>0.05)	0.06
Mn	Format	0.7	n.s. (>0.05)	0.03
	Time	15.0	< 0.001	0.58
	Format×Time	0.5	n.s. (>0.05)	0.02

The brewing time factor clearly exhibited the largest influence on mineral release, as reflected by its high *F*-values and large η^2^ in Table 7. This finding agrees with reports that longer steeping enhances the leaching of minerals from tea leaves (10, 55, 62). The effect of tea format was comparatively smaller, but still notable for specific elements: loose-leaf tea yielded significantly higher Fe, Zn, Mg, and Cu concentrations than bagged tea. These results are in line with earlier studies showing that bagged teas often contain lower levels of certain minerals than loose leaves, likely due to the finer particles and processing of tea bags (63–65). Finer tea particles can increase surface area yet may originate from lower-grade material or lose mineral-rich dust, which can reduce mineral content in the infusion (66). Importantly, the lack of any interaction effect means that extending brewing time benefits mineral extraction regardless of format. In practical terms, this suggests that prolonging infusion time is a more critical factor for maximizing mineral release from black tea than the choice between loose-leaf or bagged format. Overall, our results underscore that while using loose leaves can modestly improve the extraction of certain nutrients, brew duration is the dominant factor controlling mineral yield in tea infusions, consistent with the literature on tea preparation and mineral availability (66, 67).

Limitations and future perspectives. Although the brewing conditions applied here provide a standardized basis for comparing tea matrix and brewing format, only steeping time was systematically varied. Other preparation variables that can influence mineral leaching and post-digestion bioaccessibility, such as water temperature, tea to water ratio, and the number of infusions (re-steeping), were not examined. Therefore, extrapolation of the present findings to the full diversity of real-world consumer practices should be made with caution. Future studies should employ factorial designs and response surface methodology (DoE/RSM) to jointly model and optimize these parameters using mineral leaching efficiency and INFOGEST based bioaccessibility as response variables. Additionally, the study did not characterize trace-element speciation or quantify binding/stability constants of mineral–ligand complexes in dry teas or infusions; such questions require dedicated speciation approaches (e.g., chromatographic separation coupled to element-specific detection or spectroscopic methods) beyond the present scope (17–19). Also, future studies should quantify total phenolics (e.g., Folin–Ciocalteu) and profile major subclasses by chromatographic methods (e.g., HPLC-DAD/LC–MS) alongside *in-vitro* bioaccessibility to link matrix polyphenols with element-specific dialyzability (53).

## 4 Conclusion

This study demonstrated that mineral bioaccessibility from tea is not determined solely by total elemental content but is strongly modulated by matrix composition, infusion duration, and physical format. Rosehip tea emerged as the most effective medium for enhancing the bioaccessibility of nutritionally critical elements such as Fe and Zn, owing to its acidic pH and high organic acid content, whereas *Camellia sinensis* teas showed lower values, likely due to polyphenol-mediated chelation. Notably, Mg bioaccessibility was highest in green tea, indicating that matrix effects were element-dependent (e.g., Mg bioaccessibility: 61.92% in green tea vs. 54.04% in rosehip tea). Brewing time also played a critical role: while short infusions were sufficient for monovalent ions such as K, extended steeping up to 10–15 min improved the release of divalent minerals, though with diminishing returns after 10 min (*p* < 0.05). Loose-leaf teas consistently yielded higher levels of Cu, Fe, Mg, and Zn than bagged teas, underscoring the influence of tea format. When expressed on a per-serving basis (200 mL, 15 min), the bioaccessible fraction corresponded to 0.13–0.51 mg Fe/cup, 1.46–3.49 mg Ca/cup, and 10.39–14.20 mg K/cup, whereas Zn reached 3.49–6.06 mg/cup depending on tea type. Overall, these serving-level data indicate that tea infusions contribute modestly to Fe, Ca, and K intakes but may provide quantitatively relevant amounts of Zn (and, to a lesser extent, Cu and Mn) within the bioaccessible fraction under the tested conditions.

Future research should aim to integrate polyphenol and organic acid profiling (e.g., via LC–MS/MS or metabolomics approaches) with *in-vitro* and *in-vivo* absorption models to better elucidate mineral–matrix interactions. Expanding this work to include variability across tea brands, origins, and processing methods will also strengthen dietary recommendations. Ultimately, clarifying how tea’s biochemical composition governs mineral solubility and post-digestion availability may support more evidence-based consumer guidance and formulation strategies. In addition, DoE/RSM-based optimization of brewing parameters (temperature, tea-to-water ratio, and re-steeping) would help translate comparative findings into practical preparation guidance.

## Data Availability

The original contributions presented in the study are included in the article/supplementary material, further inquiries can be directed to the corresponding author.
