# Correction: Mineral infusion and *in-vitro* bioaccessibility in *Camellia sinensis* and herbal tea: influence of matrix and brewing format

**DOI:** 10.3389/fnut.2026.1875386

**Published:** 2026-05-15

**Authors:** Hakan Apaydın

**Affiliations:** Scientific and Technical Application and Research Center, North Campus, Hitit University, Çorum, Türkiye

**Keywords:** brewing time, *Camellia sinensis*, herbal teas, mineral bioaccessibility, tea infusion

In the published article, there was a mistake in Reference 58. The access date of the online source was erroneously written as “Accessed October 5, 2026”. The correct access date is “Accessed October 5, 2025”.

The reference for National Institutes of Health Calcium (2023) was erroneously written as:

58. National Institutes of Health Calcium (2023). Strengthening knowledge and understanding of dietary supplements. Available online at: https://ods.od.nih.gov/factsheets/Calcium-Consumer/ (Accessed October 5, 2026).

It should be:

58. National Institutes of Health Calcium. *Strengthening Knowledge and Understanding of Dietary Supplements*. (2023). Available online at: https://ods.od.nih.gov/factsheets/Calcium-Consumer/ (Accessed October 5, 2025).

The original version of this article has been updated.

